# An Adaptive Supervisory Sliding Fuzzy Cerebellar Model Articulation Controller for Sensorless Vector-Controlled Induction Motor Drive Systems

**DOI:** 10.3390/s150407323

**Published:** 2015-03-25

**Authors:** Shun-Yuan Wang, Chwan-Lu Tseng, Shou-Chuang Lin, Chun-Jung Chiu, Jen-Hsiang Chou

**Affiliations:** Department of Electrical Engineering, National Taipei University of Technology, No. 1, Sec. 3, Chung-Hsiao E. Rd., Taipei 10608, Taiwan; E-Mails: sywang@ntut.edu.tw (S.-Y.W.); lin74245@ms37.hinet.net (S.-C.L.); ranken8383@gmail.com (C.-J.C.); jhchou@ntut.edu.tw (J.-H.C.)

**Keywords:** speed sensorless vector control, fuzzy cerebellar model articulation controller (FCMAC), integral sliding surface, Lyapunov theory

## Abstract

This paper presents the implementation of an adaptive supervisory sliding fuzzy cerebellar model articulation controller (FCMAC) in the speed sensorless vector control of an induction motor (IM) drive system. The proposed adaptive supervisory sliding FCMAC comprised a supervisory controller, integral sliding surface, and an adaptive FCMAC. The integral sliding surface was employed to eliminate steady-state errors and enhance the responsiveness of the system. The adaptive FCMAC incorporated an FCMAC with a compensating controller to perform a desired control action. The proposed controller was derived using the Lyapunov approach, which guarantees learning-error convergence. The implementation of three intelligent control schemes—the adaptive supervisory sliding FCMAC, adaptive sliding FCMAC, and adaptive sliding CMAC—were experimentally investigated under various conditions in a realistic sensorless vector-controlled IM drive system. The root mean square error (RMSE) was used as a performance index to evaluate the experimental results of each control scheme. The analysis results indicated that the proposed adaptive supervisory sliding FCMAC substantially improved the system performance compared with the other control schemes.

## 1. Introduction

Vector-controlled induction motors (IMs) have been implemented in various industrial applications [1,2,3,4]. The primary advantage of using the vector-control technique is that it guarantees that the torque and flux controls are decoupled and it is easily implemented in IM drives. However, vector-controlled IM drives are difficult to control, particularly because of their nonlinear characteristics and inherent uncertainties. Therefore, developing an effective method for designing a speed controller for high-performance vector-controlled IM drives is crucial.

In recent decades, several studies have developed intelligent methods for various applications. Such methods include neural networks (NNs) [5,6,7,8] and the cerebellar model articulation controller (CMAC) [9,10,11,12]. NNs are favorable because they exhibit excellent learning capacity and they require no human experience or previously learned physical system models. However, in general, NN learning algorithms are complex, and online machine learning is time-consuming because all weight-updating is performed during each learning cycle, which increases the computational burden of NNs. In 1975, Albus [9] presented a subclass of NNs—the CMAC—to address these problems. The CMAC exhibits numerous advantageous characteristics in comparison with other NNs (e.g., rapid learning, excellent generalization capability, and simple computation). Thus, the CMAC is more effective than NNs in real-time control applications [10,11,12]. Recently, the conventional CMAC has been improved substantially, particularly in real-time applications. Previous studies have addressed various aspects associated with the conventional CMAC, including the selection of a basis function, input-space partitioning, weight-space size, and the incorporation of appropriate learning algorithms. These aspects directly affect control performance. However, the outputs of the traditional CMAC are not continuous for consecutive quantized states, which can cause control actions to fluctuate.

The fuzzy CMAC (FCMAC) in [12,13] was designed based on the fuzzy control scheme of the conventional CMAC. The primary distinction between the FCMAC and traditional CMAC is that the FCMAC features a membership function in its mapping process; the corresponding input space is continuous, and the input value can be any value in the range [0, 1]. Hence, control performance can be enhanced by developing appropriate membership functions instead of modifying the partition size. Moreover, the FCMAC weights are adjusted online according to an adaptive law derived using the Lyapunov approach. Therefore, the output quality of the FCMAC is smoother than that of the conventional CMAC; moreover, the FCMAC retains its approximation ability, and learns rapidly.

This paper proposes an adaptive supervisory sliding FCMAC to improve the performance of the FCMAC. The proposed controller comprises a supervisory controller, integral sliding surface [14,15,16], and adaptive FCMAC. The supervisory controller monitors the overall process and continually maintains the considered errors within predefined boundaries; thus, errors occurring within a certain threshold can be controlled effectively. The integral sliding surface [17] is used when a boundary layer is introduced around the sliding surface to eliminate the steady-state error that results from a continual approaching process of the switching control. Additionally, the adaptive FCMAC combines the FCMAC with a compensating controller that is used for learning and approximating the system dynamics. The advantages of the adaptive supervisory sliding FCMAC include rapid online learning, universal approximation capability, and robustness against external disturbances and uncertainties. To the authors’ knowledge, there are few literatures about the design and applications of adaptive supervisory sliding FCMAC to the induction motor drives. To confirm the effectiveness and practicability of the proposed adaptive supervisory sliding FCMAC, it was adopted as a speed controller in a sensorless vector-controlled IM drive system. Simulations and experiments were conducted to compare the performance of the proposed controller with that of other controllers. The simulation results confirmed the robustness of the proposed controller under conditions of fluctuating IM parameters, including the stator resistance, rotor resistance, rotor inertia, and viscous frictional coefficient. In the experiments, the implementation of three intelligent control schemes—the adaptive supervisory sliding FCMAC, adaptive sliding FCMAC, and adaptive sliding CMAC—was examined in a practical vector-controlled IM drive system. To compare their performance, the root mean square error (RMSE) performance index was used to evaluate the experimental results. The analysis results indicated that the proposed adaptive supervisory sliding FCMAC markedly outperformed the other two intelligent controllers.

The remainder of this paper is organized as follows: Section 2 introduces the vector-controlled IM system; Section 3 presents the design of the proposed the adaptive supervisory sliding FCMAC; Section 4 details the simulations and experiments conducted for comparison; and Section 5 offers a conclusion.

## 2. Induction Motor Vector Control System

A nonlinear three-phase Y-connected squirrel-cage IM in a synchronously rotating reference frame can be expressed using the following equations [1,2,3,4]:

(1) $$(R_{s}+\sigma L_{s}p)i_{ds}^{e}-\omega_{e}\sigma L_{s}i_{qs}^{e}+\frac{L_{m}}{L_{r}}p\lambda_{dr}^{e}-\omega_{e}\frac{L_{m}}{L_{r}}\lambda_{qr}^{e}=v_{ds}^{e}$$

(2) $$\omega_{e}\sigma L_{s}i_{ds}^{e}+(R_{s}+\sigma L_{s}p)i_{qs}^{e}+\omega_{e}\frac{L_{m}}{L_{r}}\lambda_{dr}^{e}+\frac{L_{m}}{L_{r}}p\lambda_{qr}^{e}=v_{qs}^{e}$$

(3) $$-R_{r}L_{m}i_{ds}^{e}+(R_{r}+L_{r}p)\lambda_{dr}^{e}-\omega_{sl}L_{r}\lambda_{qr}^{e}=0$$

(4) $$-R_{r}L_{m}i_{qs}^{e}+\omega_{sl}L_{r}\lambda_{dr}^{e}+(R_{r}+L_{r}p)\lambda_{qr}^{e}=0$$

where $v_{ds}^{e}$, $v_{qs}^{e}$, $i_{ds}^{e}$, $i_{qs}^{e}$, $\lambda_{dr}^{e}$, and $\lambda_{qr}^{e}$ denote the *d*-axis and *q*-axis stator voltages, *d*-axis and *q*-axis stator currents, and rotor fluxes, respectively; *R_s_*, *R_r_*, *L_s_*, *L_r_*, and *L_m_* are the stator resistances, rotor resistances, stator inductances, rotor inductances, and mutual inductances, respectively; ω*_sl_* = ω*_e_* − ω*_r_* is the rotor slip speed; ω*_r_* and ω*_e_* are the rotor and electrical speeds, respectively; σ = 1 − *L*2 *m*/*L_s_L_r_* is the leakage coefficient; and *P* and *p* denote the number of poles and the differential operator, respectively.

According to vector control theory, the rotor flux is aligned with the *d^e^*-axis, the *q^e^*-axis rotor flux is zero, and the magnetizing current $i_{ds}^{e}$ is maintained at the rated value. Thus, the torque-producing current component can be adjusted according to the load torque demand. The vector-control technique requires the rotor flux to remain oriented along the *d^e^*-axis. Accordingly, the rotor slip speed must satisfy the following condition:

(5) $$\omega_{sl}=\frac{R_{r}i_{qs}^{e}}{L_{r}i_{ds}^{e}}$$

After derivation, the *d^e^*-axis rotor flux and electromagnetic torque *T_e_* equations can be rewritten as:

(6) $$\lambda_{dr}^{e}=L_{m}i_{ds}^{e}$$

(7) $$T_{e}=\frac{3}{2}\frac{P}{2}\frac{L_{m}}{L_{r}}\lambda_{dr}^{e}i_{qs}^{e}$$

**Figure 1:**
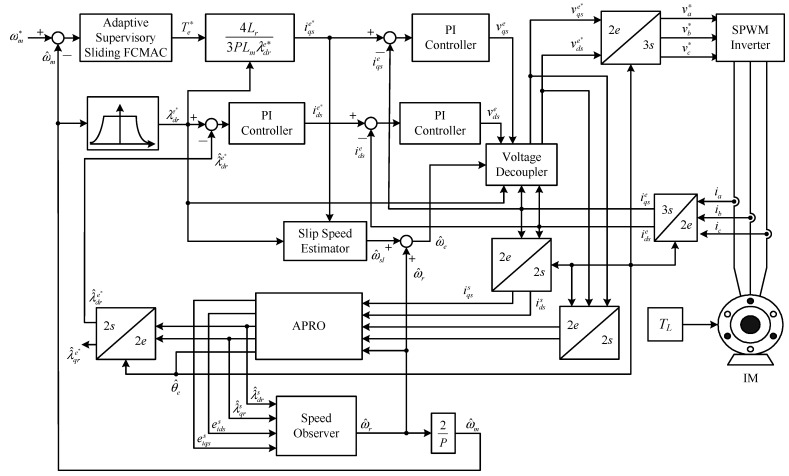
Block diagram of IM vector control system.

Equations (6) and (7) show that $\lambda_{dr}^{e}$ can be controlled according to $i_{ds}^{e}$; in other words, if $\lambda_{dr}^{e}$ is fixed, then *T_e_* can be controlled according to $i_{qs}^{e}$. By decoupling the flux current and torque current, the IM control is similar to a dc motor control. Figure 1 showed a block diagram of the IM vector control system. This structure generally requires speed- and flux-control loops. The proposed speed-control loop uses a speed observer [18,19,20] that is assumed to estimate the rotor speed eventually. Subsequently, the estimated value is returned to the speed controller—the adaptive supervisory sliding FCMAC—to generate the torque command according to the speed error. A sinusoidal pulse-width modulation (SPWM) inverter generates the inverter input signal. Moreover, the flux-control loop acquires information from the rotor flux observer. Based on mandatory results automata theory [18,19,20], an adaptive pseudo reduced-order flux observer (APRO) and the speed observer were used to obtain estimates of the rotor flux and rotor speed signal, respectively.

## 3. Design of the Adaptive Supervisory Sliding FCMAC

**Figure 2:**
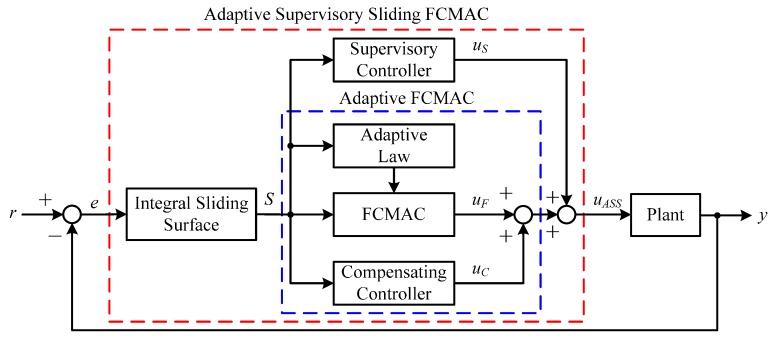
Adaptive supervisory sliding FCMAC control system block diagram.

This study presents an adaptive supervisory sliding FCMAC as a speed controller that was applied to the proposed vector-controlled IM system. The proposed adaptive supervisory sliding FCMAC control system comprises a supervisory controller, adaptive FCMAC subsystem, and integral sliding surface [17], as shown in Figure 2. The supervisory controller monitors the overall process and continually maintains the considered errors within predefined boundaries. Thus, the errors can be effectively controlled within a certain threshold. The integral sliding surface is used when a boundary layer is introduced around the sliding surface to eliminate the steady-state error that results from a continual approaching process of the switching control. In this study, the integral type sliding surface which consists of the speed tracking error and integral term. Additionally, the adaptive FCMAC subsystem combines the FCMAC with a compensating controller, which is used for learning and approximating the system dynamics. Therefore, the advantages of the adaptive supervisory sliding FCMAC include rapid online learning, universal approximation capability, and robustness to external disturbance and uncertainty. The sliding surface variable *S* denotes the input signal of the adaptive FCMAC and supervisory controller. The integral type sliding surface used in this study is defined in Equation (8), comprising the speed-tracking error and integral term:

(8) $$S(t)=e(t)+\int_{0}^{t}Qe(\tau )d\tau$$

where *Q* is a positive constant. Additionally, the adaptive FCMAC subsystem comprises an FCMAC and compensating controller. The control output *u_ASS_* of the adaptive supervisory sliding FCMAC can thus be formulated as:

(9) $$u_{ASS}=u_{S}+u_{F}+u_{C}$$

where *u_S_* is the supervisory control quantity; *u_F_* and *u_C_* denote the FCMAC and compensating control quantities output from the adaptive FCMAC, respectively; and *r* and *y* are the input and output of the control system, respectively. The details of the adaptive supervisory sliding FCMAC design are addressed in the sequel.

### 3.1. Supervisory Controller Design

The supervisory controller was designed to provide additional control quantities for large tracking errors. Consider the mechanical equation of IM drives expressed as:

(10) $${\dot{\omega }}_{m}=A_{C}\omega_{m}+B_{C}(T_{e}-T_{L})+\zeta$$

where *A_C_* = −*B_m_*/*J_m_*, *B_C_* = 1/*J_m_*, *T_e_* is the electromagnetic torque, *T_L_* denotes the load torque, *J_m_* represents the rotor inertia, and *B_m_* is the viscous frictional coefficient. The term ζ denotes the unmodeled dynamics satisfying |ζ| ≤ *h*, where *h* is the upper bound of ζ and ω*_m_* is the rotor speed of the IM. The term *T_e_* serves as the control input *u_ASS_* in Equation (9); accordingly, Equation (10) can be rewritten as:

(11) $${\dot{\omega }}_{m}=A_{C}\omega_{m}+B_{C}(u_{ASS}-T_{L})+\zeta$$

In practical applications, the real parameters change when the IM drive operates under various conditions. Consequently, the IM parameters contain nominal and variation values. Thus, Equation (11) can be rewritten as:

(12) $${\dot{\omega }}_{m}=A_{C}\omega_{m}+B_{C}(u_{ASS}-T_{L})+\zeta =({\bar{A}}_{C}+\Delta A_{C})\omega_{m}+({\bar{B}}_{C}+\Delta B_{C})(u_{ASS}-T_{L})+\zeta ={\bar{A}}_{C}\omega_{m}+{\bar{B}}_{C}u_{ASS}+\Delta A_{C}\omega_{m}-{\bar{B}}_{C}T_{L}+\Delta B_{C}(u_{ASS}-T_{L})+\zeta ={\bar{A}}_{C}\omega_{m}+{\bar{B}}_{C}u_{ASS}+\zeta_{1}$$

where ${\bar{A}}_{C}$ and ${\bar{B}}_{C}$ are the nominal values of *A_C_* and *B_C_*, respectively; Δ*A_C_* and Δ*B_C_* are variation values; and ζ_1_ is the system uncertainty, which comprises the magnitude of parameter variations, external load disturbances, and unmodeled dynamics. If the nominal values and system uncertainty upper bound of the IM are known, an ideal control law u^*^ can be defined as follows:

(13) $$u^{*}=\frac{1}{{\bar{B}}_{C}}[-{\bar{A}}_{C}\omega_{m}-\zeta_{1}+{\dot{\omega }}_{m}^{*}+k_{1}e]$$

where *k*_1_ is a positive constant, and $e=\omega_{m}^{*}-\omega_{m}$ is the speed-tracking error for the rotor. Substituting u* into Equation (12) to replace *uASS* results in $\dot{e}+k_{1}e=0$. Because *k*1 is a positive value, $\dot{e}+k_{1}e=0$ is stable, implying that the rotor speed can asymptotically track the desired trajectory of the rotor speed command. Although the nominal values of the IM parameters can be measured, the parameter variations and external load disturbance are initially unknown. Therefore, the ideal control law u^*^ in Equation (13) is practically impossible. To overcome this difficulty, the output of the adaptive supervisory sliding FCMAC (*i.e.*, *u_ASS_*) is used to approximate the ideal control law u^*^, as expressed in Equation (9). Substituting Equations (9) and (13) into Equation (12) yields:

(14) $$\dot{e}=-k_{1}e+{\bar{B}}_{C}(u^{*}-u_{S}-u_{F}-u_{C})$$

Differentiating Equation (8) relative to time yields:

(15) $$\dot{S}=\dot{e}+Qe$$

Substituting Equation (14) into Equation (15) yields:

(16) $$\dot{S}=-k_{1}e+{\bar{B}}_{C}(u^{*}-u_{S}-u_{F}-u_{C})+Qe$$

To examine the stability of Equation (16), a Lyapunov function candidate *V_S_* is considered as:

(17) $$V_{S}=\frac{1}{2}S^{2}$$

Differentiating the Lyapunov function in Equation (17) yields:

(18) $$\begin{array}{l} {\dot{V}}_{S}=S\dot{S}\\ \;=S[-k_{1}e+{\bar{B}}_{C}(u^{*}-u_{S}-u_{F}-u_{C})+Qe]\\ \;\leq -Sk_{1}e+\left|S{\bar{B}}_{C}\right|\left(\left|u^{*}\right|+\left|u_{C}+u_{F}\right|\right)-S{\bar{B}}_{C}u_{S}+SQe \end{array}$$

Substituting Equation (13) into Equation (18) yields:

(19) $${\dot{V}}_{S}\leq -Sk_{1}e+\left|S{\bar{B}}_{C}\right|[\frac{1}{{\bar{B}}_{C}}\left(\left|{\bar{A}}_{C}\omega_{m}\right|+\left|\zeta_{1}\right|+\left|{\dot{\omega }}_{m}^{*}\right|+\left|k_{1}e\right|\right)+\left|u_{C}+u_{F}\right|]-S{\bar{B}}_{C}u_{S}+SQe$$

To satisfy ${\dot{V}}_{S}<0$, the supervisory control effort *u_S_* is designed as follows:

(20) $$u_{S}=I\mathrm{sgn}(S{\bar{B}}_{C})\left[\left|u_{C}+u_{F}\right|+\frac{1}{{\bar{B}}_{C}}\left(h_{2}(\omega_{m})+h_{1}+\left|{\dot{\omega }}_{m}^{*}\right|+\left|k_{1}e\right|+\left|(k_{1}Q-Q^{2})\int_{0}^{t}e(\tau )d\tau \right|\right)\right]$$

where *h*2(ω*m*) is the upper bound function of $\left|{\bar{A}}_{C}\omega_{m}\right|$ and *h*_1_ is the upper bound of |ζ_1_|. In addition, sgn(·) is the sign function, and *I* is a switching operator, which is defined as:

(21) $$I=\{\begin{array}{c} 1,\text{ if }V_{S}\geq D^{U}\\ 0,\text{ if }V_{S}<D^{U} \end{array}$$

where *D^U^* > 0 is the specified boundary. Let *Q* < *k*_1_, then select *I* = 1, and substitute Equation (20) into Equation (19) to yield:

(22) $${\dot{V}}_{S}\leq -S^{2}(k_{1}-Q)<0$$

Equation (22) showed that the supervisory control effort given by Equation (20) makes ${\dot{V}}_{S}<0$ for *V_S_* ≥ *D^U^*, and tracking error *e* is not equal to zero. Thus, the error is effectively controlled within the bounded range.

### 3.2. Adaptive Fuzzy Cerebellar Model Articulation Controller Subsystem

#### 3.2.1. Fuzzy Cerebellar Model Articulation Controller Subsystem

**Figure 3:**
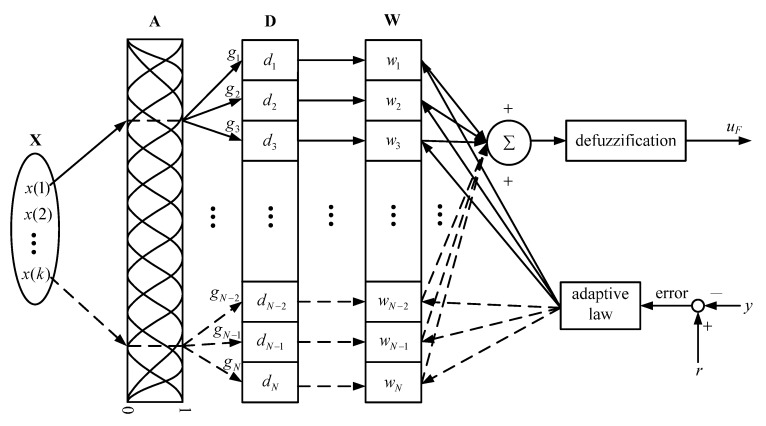
Operational concept of the FCMAC.

Figure 3 depicts the design of the proposed FCMAC that integrates the conventional CMAC and fuzzy scheme. This control structure consists of the input space **X**, membership function space **A**, receptive field space **D**, weight memory space **W**, and adaptive law. The input signals in **X** are measured and fuzzified appropriately and then mapped to **A**. Each input value is transformed into a “firing strength” based on the corresponding Gaussian membership functions. Each membership function can be formulated as:

(23) $$g_{i}=\exp\left[\frac{-{\left(x(k)-m_{i}\right)}^{2}}{\sigma_{i}^{2}}\right]\mathrm{for}i=1,2,\ldots ,N$$

where $g_{i}$ is the firing strength of the *i*th membership function in **A**; *x*(*k*) is the fuzzified input value at the *k*th sampling epoch; $m_{i}$ and $\sigma_{i}$ are the mean and variance of the *i*th Gaussian function, respectively; and *N* is the number of Gaussian membership functions.

Each output of the membership function is thus connected to a single element of **D**. Each element of **D** generates a one-to-one mapping to each element of **W**. Finally, the sum of the weight values yields the FCMAC output through a defuzzification procedure. Using the center average defuzzifier, the control quantity *u_F_* of the FCMAC can be determined by computing:

(24) $$u_{F}=\frac{\sum_{i=1}^{N}g_{i}d_{i}w_{i}}{\sum_{i=1}^{N}g_{i}}=\frac{1}{\sum_{i=1}^{N}g_{i}}\left[\begin{array}{cccc} f_{1} & f_{2} & \cdots & f_{N} \end{array}\right]\;\left[\begin{array}{c} w_{1}\\ w_{2}\\ \vdots \\ w_{N} \end{array}\right]=bF^{T}W$$

where $b=1/\sum_{i=1}^{N}g_{i}$, $F={\left[\begin{array}{cccc} f_{1} & f_{2} & \cdots & f_{N} \end{array}\right]}^{T}$, $f_{i}=g_{i}d_{i}$, and $W={\left[\begin{array}{cccc} w_{1} & w_{2} & \cdots & w_{N} \end{array}\right]}^{T}$ is the weight memory vector of the FCMAC. The elements of the weight memory space are updated according to the adaptive law derived from Lyapunov theory. Each weight is initialized with a zero value before performing the adjustment. This process repeats until the output error converges in the permitted range. The convergence of the inferential process and stability of the FCMAC are discussed in the following section.

#### 3.2.2. Stability Analysis of the Adaptive Fuzzy Cerebellar Model Articulation Controller Subsystem

Assuming that a reconstruction error ρ is defined as:

(25) $$\rho =u^{*}-u_{F}^{*}$$

where $u_{F}^{*}$ is the ideal control output of the FCMAC which is used to minimize the gap between u* and *uASS*. In this paper, ρ is assumed to be less than a positive constant γ (*i.e.*, |ρ|≤γ). The control law of the FCMAC is given in Equation (24). Substituting Equations (24) and (25) into Equation (14) yields:

(26) $$\dot{e}=-k_{1}e+{\bar{B}}_{C}(\rho +bF^{T}\tilde{W}-u_{S}-u_{C})$$

where $\tilde{W}=W^{*}-W$, and $W^{*}$ is an adjustable optimal-weight memory vector of the FCMAC. Substituting Equation (26) into Equation (15) yields:

(27) $$\dot{S}=-k_{1}e+{\bar{B}}_{C}(\rho +bF^{T}\tilde{W}-u_{S}-u_{C})+Qe$$

A Lyapunov function candidate *V_C_* can be defined as:

(28) $$V_{C}=\frac{1}{2}S^{2}+\frac{1}{2\beta }{\tilde{W}}^{T}\tilde{W}$$

where β is the FCMAC learning rate. Differentiating Equation (28) relative to time yields:

(29) $$\begin{array}{l} {\dot{V}}_{C}=S\dot{S}-\frac{1}{\beta }{\tilde{W}}^{T}\dot{W}\\ \;=S\left[-k_{1}e+{\bar{B}}_{C}(\rho +bF^{T}\tilde{W}-u_{S}-u_{C})+Qe\right]-\frac{1}{\beta }{\tilde{W}}^{T}\dot{W}\\ \;=-Sk_{1}e+S{\bar{B}}_{C}\rho +S{\bar{B}}_{C}bF^{T}\tilde{W}-S{\bar{B}}_{C}u_{S}-S{\bar{B}}_{C}u_{C}+SQe-\frac{1}{\beta }{\dot{W}}^{T}\tilde{W} \end{array}$$

According to Equation (29), the adaptive law ${\dot{W}}^{T}$ of the FCMAC can be derived as:

(30) $${\dot{W}}^{T}=\beta S{\bar{B}}_{C}bF^{T}$$

Substituting Equation (30) into Equation (29) yields:

(31) $${\dot{V}}_{C}=-Sk_{1}e+S{\bar{B}}_{C}\rho -S{\bar{B}}_{C}u_{S}-S{\bar{B}}_{C}u_{C}+SQe$$

Because $S{\bar{B}}_{C}u_{S}>0$, Equation (31) can be rewritten as:

(32) $${\dot{V}}_{C}\leq -Sk_{1}e+\left|S{\bar{B}}_{C}\right|\left|\rho \right|-S{\bar{B}}_{C}u_{C}+SQe$$

To satisfy ${\dot{V}}_{C}\leq 0$, the compensating control *u_C_* is expressed as:

(33) $$u_{C}=\gamma \mathrm{sgn}(S{\bar{B}}_{C})+\frac{1}{{\bar{B}}_{C}}(k_{1}Q-Q^{2})\int_{0}^{t}e(\tau )d\tau$$

Because $Q<k_{1}$, substituting Equation (33) into Equation (32) yields:

(34) $${\dot{V}}_{C}\leq -S^{2}(k_{1}-Q)\leq 0$$

Because ${\dot{V}}_{C}\leq 0$, $V_{C}(t)\leq V_{C}(0)$, which implies that *S* and $\tilde{W}$ are bounded. Consider the function $\Xi =S^{2}(k_{1}-Q)\leq -{\dot{V}}_{C}$ and integrate the function Ξ relative to time. Accordingly:

(35) $$\int_{\;0}^{\;t}\Xi (\tau )d\text{τ}\leq V_{C}(0)-V_{C}(t)$$

Because *V_C_* (0) is bounded and *V_C_* (*t*) is nonincreasing and bounded, the results are:

(36) $$\underset{t\to \infty }{\lim}\int_{\;0}^{\;t}\Xi (\tau )d\tau <\infty$$

In addition, differentiating the function $\Xi =S^{2}(k_{1}-Q)\leq -{\dot{V}}_{C}$ relative to time yields $\dot{\Xi }=2S\dot{S}(k_{1}-Q)$, which is bounded. Therefore, Ξ(t) is uniformly continuous. The Barbalat lemma [15] can be used to prove that $\underset{t\to \infty }{\lim}\;\Xi (t)=0$. Consequently, e→0 when t→∞; therefore, the stability of the proposed adaptive FCMAC is guaranteed.

## 4. Simulation and Experimental Results

**Figure 4:**
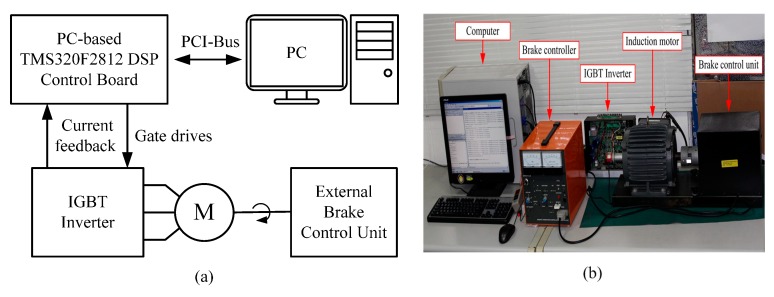
Hardware configuration for experimentation of the IM drive: (**a**) Block diagram of the experimental platform; (**b**) Platform photograph.

The practicability and robustness of the proposed adaptive supervisory sliding FCMAC were verified by conducting simulations and experiments of the speed controller of the vector-controlled IM system (Figure 1). Figure 4 shows a block diagram and photograph of the experimental platform for implementing the proposed system. The experiment was conducted using a PC, a DSP PC-based control board (DSP TMS320F2812 digital control board), insulated-gate bipolar transistor inverter, 2.2-kW IM, and external brake-control unit for applying an external load. For the experimental platform, the DSP control board plugs into the PC and provides a PWM module, A/D and D/A interfaces between PC and the induction motor drive. The control algorithm encompassed the proposed adaptive supervisory sliding FCMAC, vector control loops, APRO, and speed observer, which were designed and implemented using MATLAB/Simulink. The sampling frequency of the experimental platform was 10 kHz. In this study, the speed command was used to perform single-quadrant operation as a spline-shaped curve rather than a step function. Table 1 shows the IM parameters of the experimental platform. Table 2 shows the parameters of the proposed adaptive supervisory sliding FCMAC. The adaptive supervisory sliding FCMAC parameters include *h*_2_(ω*m*), *h*_1_, *k*_1_, ${\bar{A}}_{C}$, and ${\bar{B}}_{C}$, which depend on the motor specifications. The other parameters—such as the predefind range of the supervisory controller *D^U^*, adaptation rate of the compensating controller γ, positive constant of the sliding surface *Q*, and learning rate of the FCMAC β—were set based on experience. In both the simulation and the experimental study, the sampling time was set to 0.1 ms for the speed control loop and current control loop which was executed over a PC. The switching frequency of the SPWM inverter used in the experiment was 10 kHz, and its rated power was 3 kW. The resolution of the shaft encoder considered in the experimental setup was 2048 counts/rev. In real applications of motor control, due to protection function of inverter module and the limitation of rated motor torque command, the system may be stalled by large control signals in the startup stage. Thus, to make the controller feasible, we modified the control *u_S_* in Equation (20) to *u_SM_* as follows:

(37) $$u_{SM}=\delta \left\{I\mathrm{sgn}(S{\bar{B}}_{C})\left[\left|u_{C}+u_{F}\right|+\frac{1}{{\bar{B}}_{C}}\left(h_{2}(\omega_{m})+h_{1}+\left|{\dot{\omega }}_{m}^{*}\right|+\left|k_{1}e\right|+\left|(k_{1}Q-Q^{2})\int_{0}^{t}e(\tau )d\tau \right|\right)\right]\right\}$$

where 0 < δ < 1, the δ is set 0.07. The results of the simulations and experiments are discussed in the following section.

**Table 1:** IM parameters.

Parameter	Unit	Value
Type	-	Squirrel-cage
Rated power	kW	2.2
Rated voltage	V	220/380 (Δ/Y)
Rated current	A	8.6/5 (Δ/Y)
Rated speed	rpm	1720
Poles (P)	-	4
Rotor inertia (*J_m_*)	kg-m^2^	0.033
Viscous friction coefficient (*B_m_*)	Nm/(rad/s)	0.00825
stator resistance	Ω	0.833
rotor resistance	Ω	0.53

**Table 2:** Parameters in adaptive supervisory sliding FCMAC.

Parameter	Value	
*h*_1_	402	
*D^U^*	experiment simulation	0.7 0.1
*k*_1_	1	
*Q*	0.02	
${\bar{A}}_{C}$	−0.25	
${\bar{B}}_{C}$	30.3	
γ	0.01	
β	experiment simulation	0.0032 0.15
*N*	12	

### 4.1. Simulation Results

In order to demonstrate the feasibility of the proposed adaptive supervisory sliding FCMAC control system in induction motor system, simulation results are presented in this section. During motor operation, changing uncertain parameters (e.g., *L_r_*, *R_s_*, *R_r_*, *J_m_*, and *B_m_*) online in the experimental platform is difficult. Thus, MATLAB/Simulink was used to conduct simulations to verify the robustness of the proposed adaptive supervisory sliding FCMAC control system against the *L_r_*, *R_s_*, *R_r_*, *J_m_*, and *B_m_* variations. To reach the aim, several simulations were considered: (1) Induction motor parameters with/without incremental variation; (2) Adaptive supervisory sliding FCMAC control system; (3) Speed tracking using various operations and external load disturbance in the steady state.

**Figure 5:**
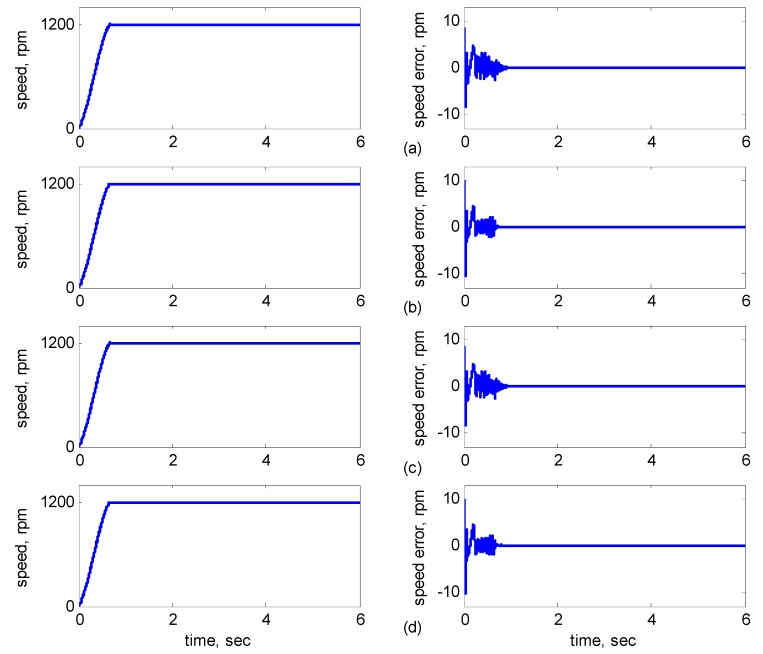
Simulation results of speed reference at 1200 rpm: (**a**) No parameter variation. (**b**) 40% incremental variation in *J_m_*; (**c**) 50% incremental variation in *B_m_*; (**d**) 40% incremental variation in *J_m_* and 50% incremental variation in *B_m_*.

**Figure 6:**
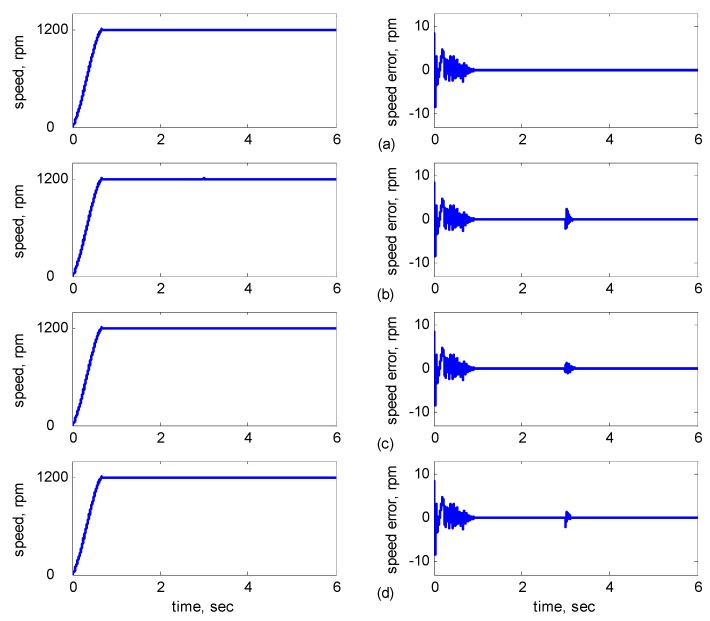
Simulation results of speed reference at 1200 rpm: (**a**) No parameter variation. (**b**) 30% incremental variation in *R_r_* at 3 s; (**c**) 30% incremental variation in *R_s_* at 3 s; (**d**) 30% incremental variation at 3 s in both *R_r_* and *R_s_*.

**Figure 7:**
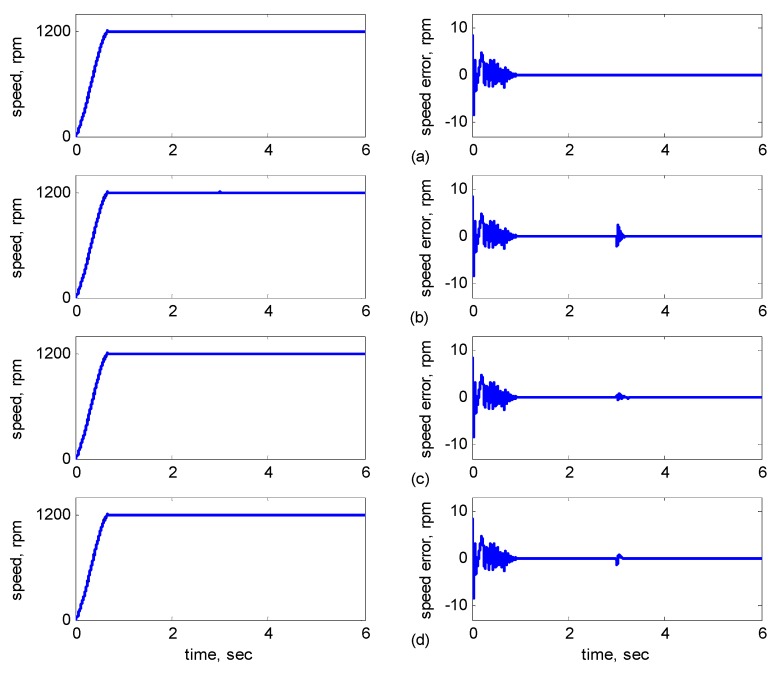
Simulation results of speed reference at 1200 rpm: (**a**) No parameter variation. (**b**) 30% incremental variation in *R_r_* at 3 s; (**c**) 10% incremental variation in *L_r_* at 3 s; (**d**) 30% incremental variation in *R_r_* and 10% incremental variation in *L_r_* at 3 s.

**Figure 8:**
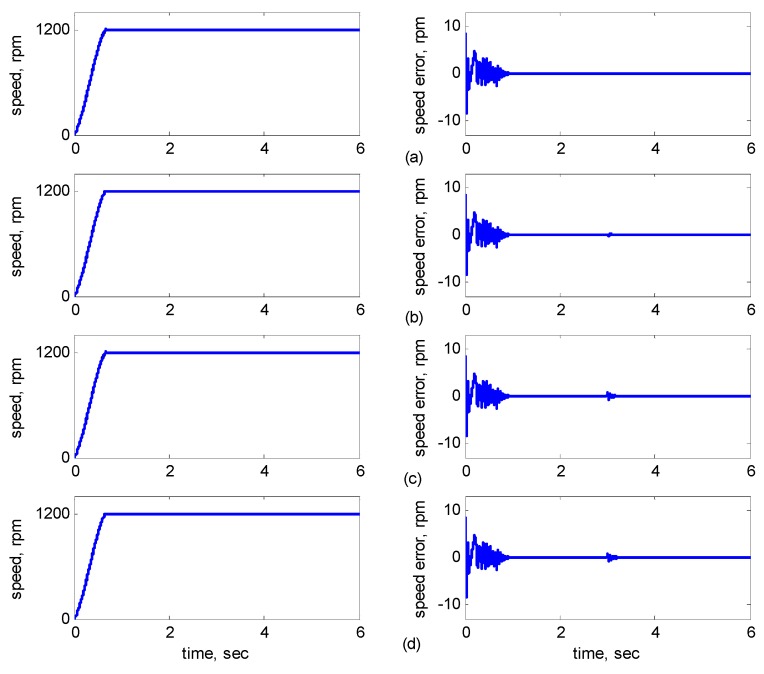
Simulation results of speed reference at 1200 rpm using the adaptive reduced order MRAS: (**a**) No parameter variation; (**b**) 30% incremental variation in *R_r_* at 3 s; (**c**) 10% incremental variation in *L_r_* at 3 s; (**d**) 30% incremental variation in *R_r_* and 10% incremental variation in *L_r_* at 3 s.

The rotor speed command was 1200 rpm with an 8-Nm torque load. Figure 5, Figure 6, Figure 7 and Figure 8 show the simulation results of parameters with/without incremental variation. Each subplot showed the responses of speed (left) and speed error (right). Figure 5a shows *J_m_* and *B_m_* without incremental variations. Figure 5b shows *J_m_* with 40% incremental variation. Figure 5c shows *B_m_* with 50% incremental variation. Figure 5d shows *J_m_* with 40% incremental variation and *B_m_* with 50% incremental variation.

Figure 6a shows *R_r_* and *R_s_* without variation at 3 s. Figure 6b shows *R_r_* with 30% variation at 3 s. Figure 6c shows *R_s_* with 30% variation at 3 s and Figure 6d shows *R_r_* and *R_s_* with 30% variation at 3 s. Figure 7 shows the dynamic responses for *R_r_* and *L_r_* with/without incremental variation at 3 s. Figure 7a shows *R_r_* and *L_r_* without variation.

Figure 7b shows *R_r_* with 30% incremental variation at 3 s. Figure 7c shows *L_r_* with 10% incremental variation at 3 s. Figure 7d shows *R_r_* with 30% and *L_r_* with 10% incremental variations at 3 s. Figure 8 shows the dynamic responses of the adaptive reduced order MRAS for *R_r_* and *L_r_* with/without incremental variation at 3 s. Figure 8a shows *R_r_* and *L_r_* without incremental variations. Figure 8b shows *R_r_* with 30% incremental variation at 3 s. Figure 8c shows *L_r_* with 10% incremental variation at 3 s. Figure 8d shows *R_r_* with 30% incremental variation at 3 s and *L_r_* with 10% incremental variation at 3 s.

As shown in Figure 5a, the maximal speed error was 8.38 rpm, and the speed error of the steady state was in the range of ±0.1 rpm. In Figure 5b–d, although the maximal speed error was 9.75 rpm when *J_m_* and *B_m_* varied, it increased by only 1.37 rpm as compared to that in Figure 5a, and the speed error of the steady state was also controlled in the range of ±0.12 rpm. In Figure 6b–d, when *R_r_* and *R_s_* varied at 3 s, the increased error range was in ±2 rpm, but in 3 s later, the error was controlled in the range of ±0.12 rpm. In Figure 7b–d, when *R_r_* and *L_r_* varied at 3 s, the increased error range was in ±2 rpm, but in 3 s later, the error was controlled in the range of ±0.12 rpm. In Figure 8b–d, when *R_r_* and *L_r_* varied at 3 s, the increased error range was in ±0.8 rpm, but in 3 s later, the error was controlled in the range of ±0.12 rpm. Therefore, the adaptive supervisory sliding FCMAC was affected only slightly when the mechanical parameters *L_r_*, *R_r_*, *R_s_*, *J_m_*, and *B_m_* were varied. Thus, the simulation results indicated the robustness of the proposed adaptive supervisory sliding FCMAC against parameter variations.

**Figure 9:**
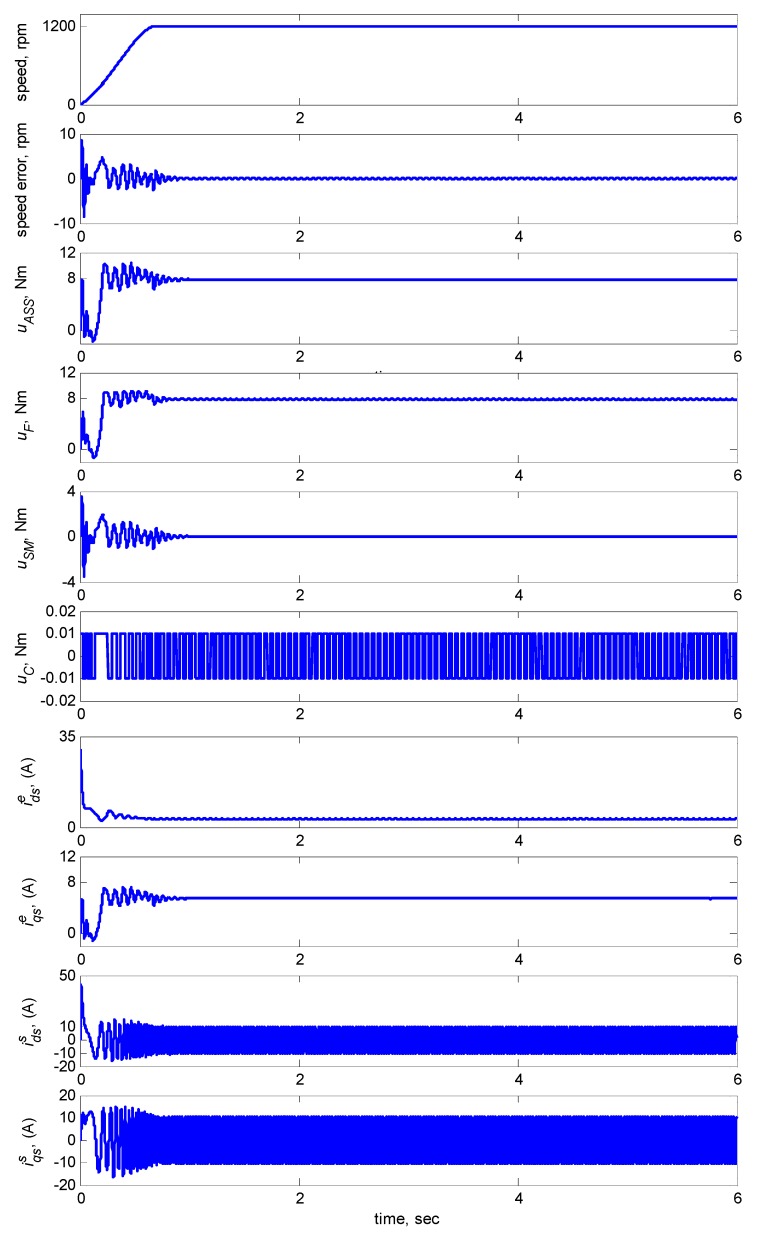
Simulation results of speed response and control efforts of adaptive supervisory sliding FCMAC at speed reference 1200 rpm with 8 Nm torque load.

Figure 9 shows the dynamic responses at 1200 rpm with an 8-Nm torque load. The subplots in Figure 9 show the responses in speed, speed error, total control law *u_ASS_*, FCMAC control *u_F_*, modified supervisory control *u_S__M_*, compensating control *u_C_*, *d-q* axis stator currents $i_{ds}^{s}$, $i_{qs}^{s}$ in the stationary reference frames and *d-q* axis stator currents $i_{ds}^{e}$, $i_{qs}^{e}$ in the synchronously rotating reference frames. Figure 9 indicated that the transient state of the speed response was relatively large when the adaptive FCMAC initiated learning. Thus, the supervisory controller provided a control quantity to improve the transient response until the learning process of the adaptive FCMAC was complete. The transient-state error was less than ±10 rpm and the speed error in the steady state was less than ±0.15 rpm.

Furthermore, to verify the tracking capability and robustness of the proposed controller against the external load, the adaptive supervisory sliding FCMAC, adaptive sliding FCMAC, and adaptive sliding CMAC were implemented for comparison. The three controllers were derived using the Lyapunov technique, adaptive control theory, and the input signal of these controllers (derived from the integral sliding surface variable). The proposed adaptive sliding CMAC designed based on the conventional CMAC equipped with adaption capability and an integral sliding surface. These controllers were operated and compared at various speeds: 1200 rpm, 2000 rpm, 36 rpm, and ±1200 rpm (four-quadrant speed regulation) with an 8-Nm torque load and an external load disturbance in the steady state of the IM system.

**Figure 10:**
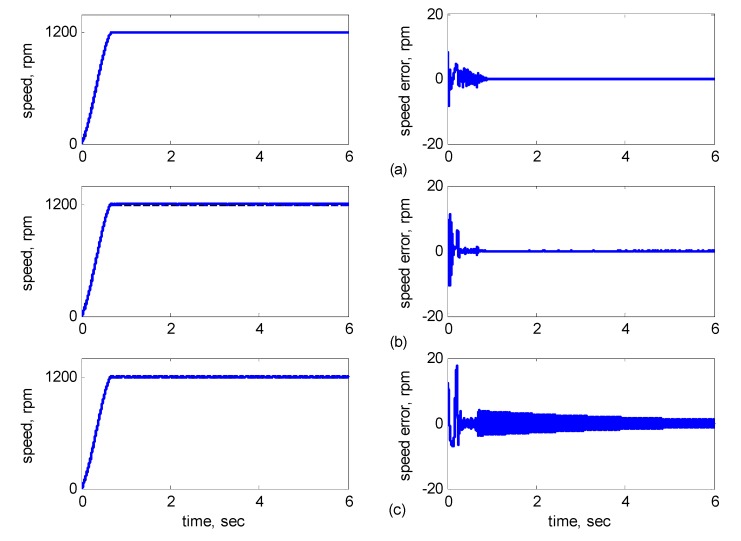
Simulation results of speed response: speed (left) and speed error (right) at speed reference 1200 rpm with 8 Nm torque load by using (**a**) Adaptive supervisory sliding FCMAC; (**b**) Adaptive sliding FCMAC; (**c**) Adaptive sliding CMAC.

**Figure 11:**
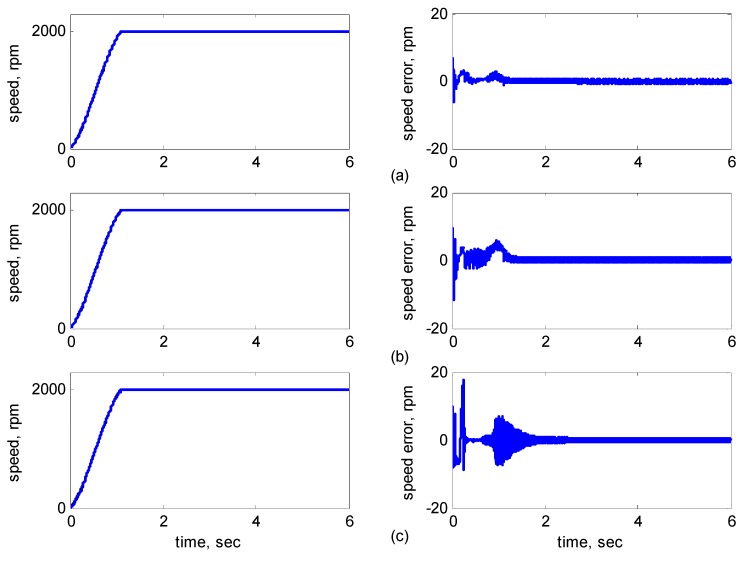
Simulation results of speed response: speed (left) and speed error (right) at speed reference 2000 rpm with 8 Nm torque load by using (**a**) Adaptive supervisory sliding FCMAC; (**b**) Adaptive sliding FCMAC; (**c**) Adaptive sliding CMAC.

**Figure 12:**
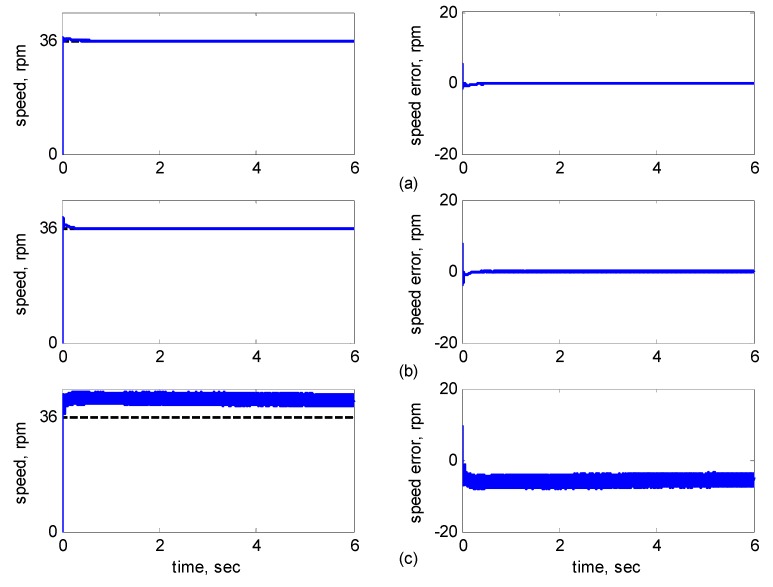
Simulation results of speed response: speed (left) and speed error (right) at speed reference 36 rpm with 8 Nm torque load by using (**a**) Adaptive supervisory sliding FCMAC; (**b**) Adaptive sliding FCMAC; (**c**) Adaptive sliding CMAC.

**Figure 13:**
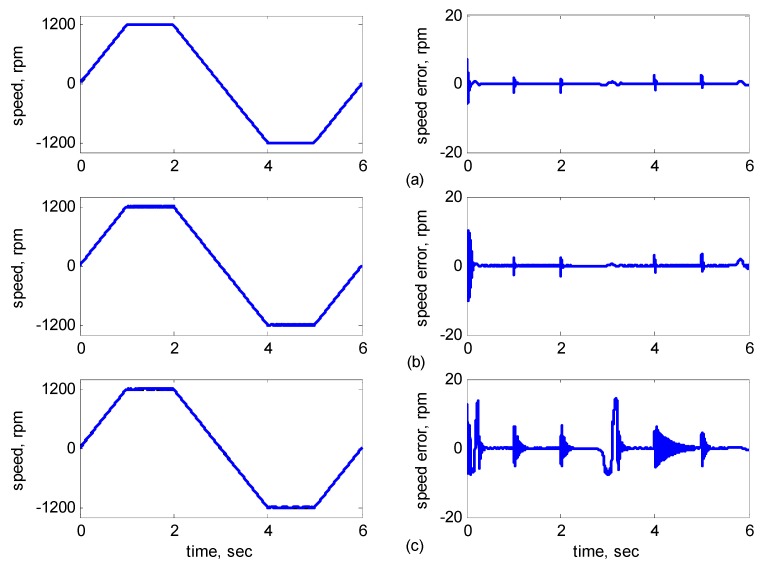
Simulation results of speed response: speed (left) and speed error (right) at speed reference ±1200 rpm with 8 Nm torque load by using (**a**) Adaptive supervisory sliding FCMAC; (**b**) Adaptive sliding FCMAC; (**c**) Adaptive sliding CMAC.

**Figure 14:**
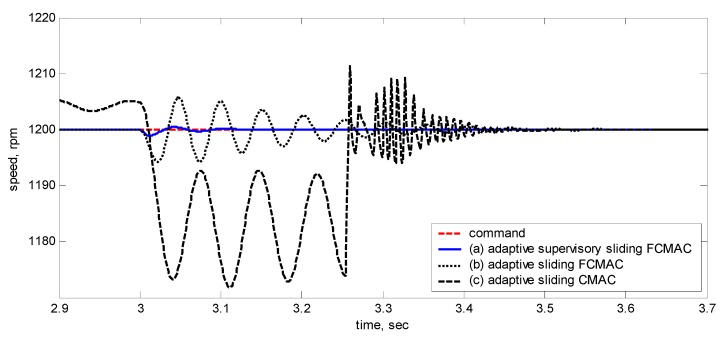
Simulation results of speed reference at 1200 rpm with 4 Nm external load disturbance applied at 3 s by using (**a**) Adaptive supervisory sliding FCMAC; (**b**) Adaptive sliding FCMAC; (**c**) Adaptive sliding CMAC.

Figure 10, Figure 11, Figure 12, Figure 13 and Figure 14 showed the simulation results. Subplot (a) presents the results using adaptive supervisory sliding FCMAC, Subplot (b) presents the results using adaptive sliding FCMAC, and Subplot (c) presents the results using adaptive sliding CMAC. The simulation results for the speed of 1200 rpm and 2000 rpm are shown in Figure 10 and Figure 11, respectively. The speed reference (2000 rpm) exceeded the rated motor speed; thus, the motor operated in the flux-weakening region. As shown in Figure 10a and Figure 11a, the tracking speed error of the adaptive supervisory sliding FCMAC was less than 0.2 rpm in the steady state. Additionally, the error was also less than that of the other two controllers in the maximal transient state under the same conditions. As shown in Figure 10b and Figure 11b, the adaptive sliding FCMAC performed well in the steady state. However, the transient state produced a large speed error and slow convergence because it lacked supervisory control. As shown in Figure 10c and Figure 11c, because the values of the association memory were either 0 or 1 when the reference state was excited in the adaptive sliding CMAC, the smoothness of the output was consequently poor and the speed fluctuated widely. Thus, compared with the other intelligent controllers, the adaptive supervisory sliding FCMAC exhibited rapid online learning in the transient state, and the output response was smoother and exhibited faster convergence in the steady state. Figure 12 shows the simulation results for the speed of 36 rpm. As shown in Figure 12a, the maximal speed error of the adaptive supervisory sliding FCMAC was 5.3 rpm in the transient state and the speed error of the steady state was within ±0.004 rpm. As shown in Figure 12b,c, the adaptive sliding FCMAC and the adaptive sliding CMAC were not equipped with supervisory controllers. The speed errors were larger than those of the adaptive supervisory sliding FCMAC in the transient state. However, compared with the responses of adaptive sliding CMAC, the adaptive sliding FCMAC still performed well in the steady state because the FCMAC produced a suitable control effort. For four-quadrant speed regulation (±1200 rpm), when the speed crossed zero, the estimation errors of the stator current increased and caused a large flux estimation error. Consequently, a speed bump occurred at the zero crossing, as shown in Figure 13. The tracking speed errors of the adaptive supervisory sliding FCMAC and adaptive sliding FCMAC were within 0.5 rpm, and that of the adaptive sliding CMAC was within 15 rpm, as indicated at the zero-crossing point. Therefore, the proposed adaptive supervisory sliding FCMAC outperformed the other two intelligent controllers in Figure 10, Figure 11, Figure 12 and Figure 13. Figure 14 showed the adaptive supervisory sliding FCMAC, adaptive sliding FCMAC, and adaptive sliding CMAC control schemes implemented separately and operated at 1200 rpm without any external load torque for 3 s. A 4-Nm load torque disturbance was applied at exactly 3 s. As shown in Figure 14, the proposed adaptive supervisory sliding FCMAC recovered the motor speed more quickly than the other two controllers did, because the supervisory controller provided a control quantity to improve the transient response. Thus, the performance of the adaptive supervisory sliding FCMAC was superior to that of the other two controllers after applying torque disturbance.

### 4.2. Experimental Results

This section presents the experimental results to verify the practicality and robustness of the proposed adaptive supervisory sliding FCMAC control system in real IMs The following experimental cases for the adaptive supervisory sliding FCMAC are discussed:

Case 1: Adaptive supervisory sliding FCMAC control system.
Case 2: Speed tracking using various operations.
Case 3: External load disturbance in the steady state.

**Figure 15:**
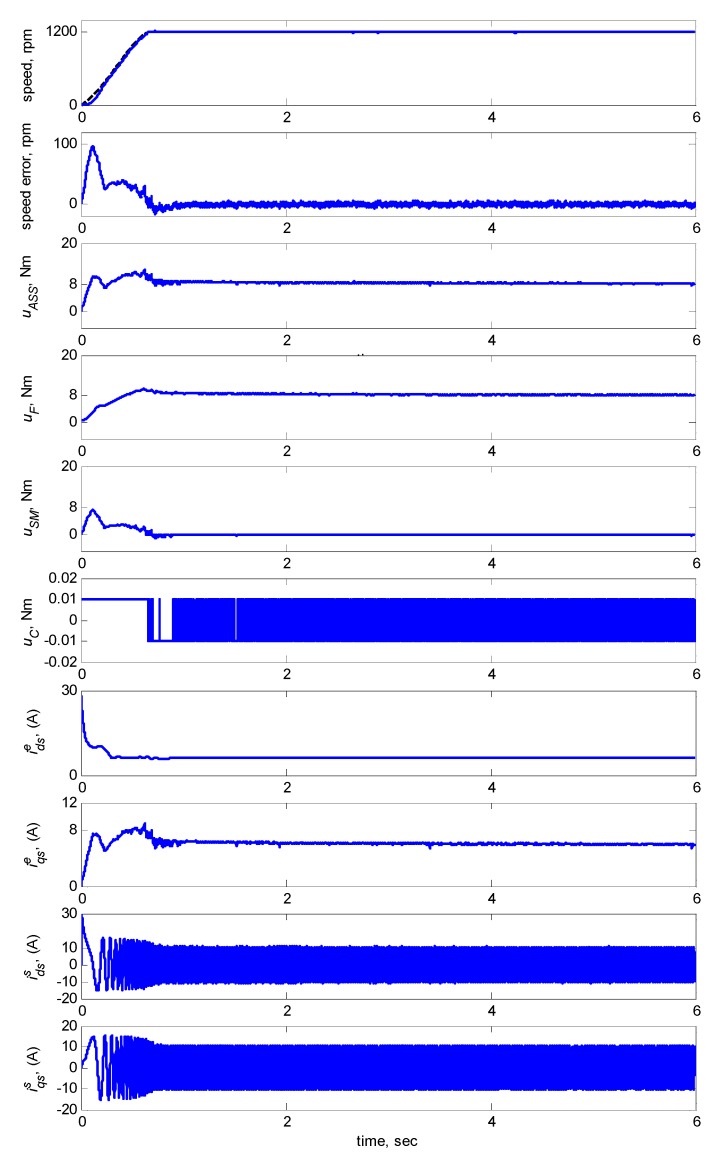
Experimental results of speed response and control efforts of adaptive supervisory sliding FCMAC at speed reference: 1200 rpm with 8 Nm torque load.

Regarding Case 1, the rotor speed was 1200 rpm with an 8-Nm torque load. The subplots in Figure 15 show the responses in speed, speed error, total control law *u_ASS_*, FCMAC control *u_F_*, supervisory control *u_S_*, and compensating control *u_C_*, *d-q* axis stator currents $i_{ds}^{s}$, $i_{qs}^{s}$ in the stationary reference frames and *d-q* axis stator currents $i_{ds}^{e}$, $i_{qs}^{e}$ in the synchronously rotating reference frames. The figure indicates that the transient state of the speed response was relatively large when the adaptive FCMAC initiated learning. Thus, the supervisory controller provided a control quantity to improve the transient response until the learning process was complete. The transient-state error was less than 100 rpm, and the speed error in the steady state was less than 5 rpm.

To verify the tracking capability and robustness of the proposed controller against the external load, the adaptive supervisory sliding FCMAC, adaptive sliding FCMAC, and adaptive sliding CMAC were implemented for comparison in Cases 2 and 3. Regarding Case 2, the three controllers were operated and compared at various speeds (*i.e.*, 1200, 2000, 36, and ±1200 rpm; four-quadrant speed regulation) with an 8-Nm torque load. An external load disturbance in the steady state of the system is presented in Case 3.

**Figure 16:**
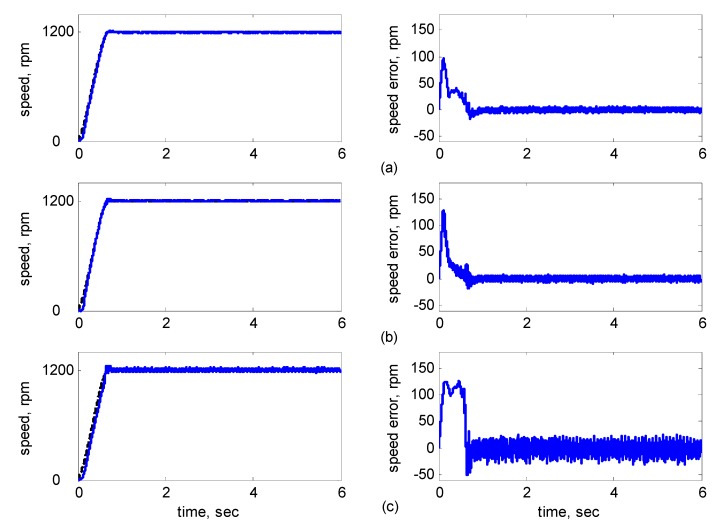
Experimental results of speed response: speed (left) and speed error (right) at speed reference 1200 rpm with 8 Nm torque load by using (**a**) Adaptive supervisory sliding FCMAC; (**b**) Adaptive sliding FCMAC; (**c**) Adaptive sliding CMAC.

**Figure 17:**
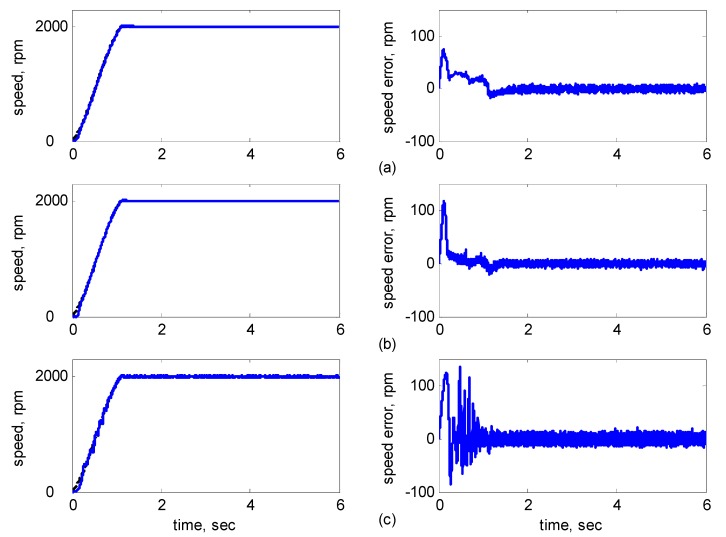
Experimental results of speed response: speed (left) and speed error (right) at speed reference 2000 rpm with 8 Nm torque load by using (**a**) Adaptive supervisory sliding FCMAC; (**b**) Adaptive sliding FCMAC; (**c**) Adaptive sliding CMAC.

**Figure 18:**
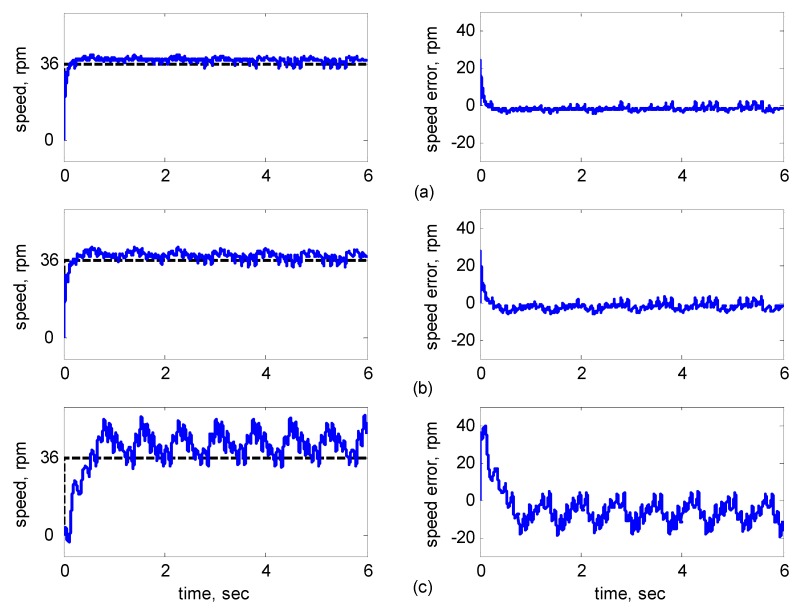
Experimental results of speed response: speed (left) and speed error (right) at speed reference 36 rpm with 8 Nm torque load by using (**a**) Adaptive supervisory sliding FCMAC; (**b**) Adaptive sliding FCMAC; (**c**) Adaptive sliding CMAC.

**Figure 19:**
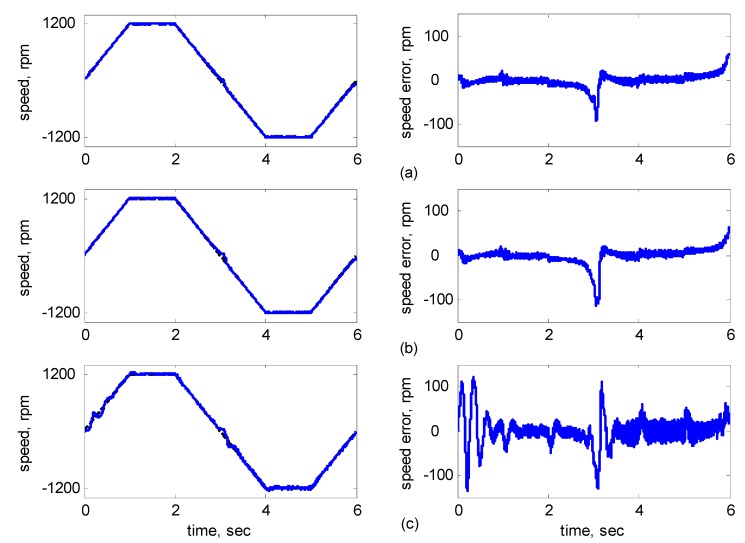
Experimental results of speed response: speed (left) and speed error (right) at speed reference ±1200 rpm with 8 Nm torque load by using (**a**) Adaptive supervisory sliding FCMAC; (**b**) Adaptive sliding FCMAC; (**c**) Adaptive sliding CMAC.

**Figure 20:**
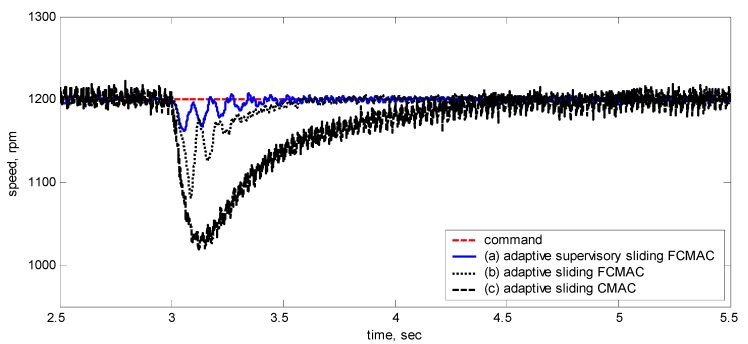
Experimental results of speed reference at 1200 rpm with 4 Nm external load disturbance applied at 3 s by using (**a**) Adaptive supervisory sliding FCMAC; (**b**) Adaptive sliding FCMAC; (**c**) Adaptive sliding CMAC.

Figure 16, Figure 17, Figure 18, Figure 19 and Figure 20 show the experimental results, where Subplot (a) presents the adaptive supervisory sliding FCMAC results, Subplot (b) depicts the adaptive sliding FCMAC results, and Subplot (c) shows the adaptive sliding CMAC results. The experimental results at 1200 and 2000 rpm are shown in Figure 16 and Figure 17, respectively. The speed reference (2000 rpm) was higher than the rated motor speed; thus, the motor operated in the flux-weakening region. As shown in Figure 16a and Figure 17a, the tracking speed error of the adaptive supervisory sliding FCMAC was less than 5 rpm in the steady state. Additionally, the error was less than that of the other two controllers in the maximal transient state under identical conditions. As shown in Figure 16b and Figure 17b, although the adaptive sliding FCMAC performed effectively in the steady state, the transient state produced a large speed error and slow convergence because no supervisory control was applied.

As indicated in Figure 16c and Figure 17c, because the values of the association memory in the adaptive sliding CMAC were either 1 or 0 when the reference state was excited, the smoothness of the output was consequently poor, and the speed fluctuated widely. Thus, compared with the other intelligent controllers, the adaptive supervisory sliding FCMAC exhibited rapid online learning in the transient state, and the output response was smoother and faster convergence was exhibited in the steady state. Thus, the proposed adaptive supervisory sliding FCMAC outperformed the other two intelligent controllers. Vector control of an IM is difficult at low speeds, particularly at 36 rpm. The interior structure of IMs inevitably causes practical dynamics. The structure diminishes the IM’s capacity to produce constant smooth torque output at low speeds, resulting in greater speed-response errors than those occurring at higher speeds. Figure 18 showed the experimental results at 36 rpm. As shown in Figure 18a, the maximal speed error of the adaptive supervisory sliding FCMAC was 24.6 rpm in the transient state, and the speed error in the steady state was within ±3 rpm. As shown in Figure 18b,c, the adaptive sliding FCMAC and adaptive sliding CMAC were not fitted with supervisory controllers. The speed errors were larger than those of the adaptive supervisory sliding FCMAC in the transient state. However, unlike the adaptive sliding CMAC, the adaptive sliding FCMAC maintained an acceptable performance level in the steady state because it produced a suitable control effort. During four-quadrant speed regulation (±1200 rpm), when the speed crossed zero, the estimation errors of the stator current increased, causing a large flux estimation error; consequently, a “speed bump” occurred when the speed was equal to zero (Figure 19). The tracking speed errors of the adaptive supervisory sliding FCMAC, adaptive sliding FCMAC, and adaptive sliding CMAC were within 95, 110, and 130 rpm, respectively (as indicated at the zero crossing). Thus, the dynamic tracking capability of the proposed adaptive supervisory sliding FCMAC enabled it to outperform the other two controllers.

Figure 20 shows the adaptive supervisory sliding FCMAC, adaptive sliding FCMAC, and adaptive sliding CMAC control schemes implemented separately and operated at 1200 rpm without any external load torque for 3 s. A 4-Nm load torque disturbance was applied at precisely the third second. The figure showed that the proposed adaptive supervisory sliding FCMAC recovered the motor speed faster than did the other two controllers because the supervisory controller provided a control quantity to improve the transient response. Thus, the performance of the adaptive supervisory sliding FCMAC was superior to that of the other two controllers after torque disturbance was applied.

This study used the RMSE as a performance index to evaluate the experimental results of each control scheme under various operating conditions. Table 3 shows the RMSE results based on 60,000 sampling points over 6 s for Case 2. The RMSE can be expressed as:

(38) $$\text{RMSE}=\sqrt{\frac{\sum_{k=1}^{L}e{\left(k\right)}^{2}}{L}}$$

where *e*(*k*) is the tracking error and *L* denotes the number of sampling points. Table 3 showed that the RMSE value of the adaptive supervisory sliding FCMAC was far lower than that of the other two control schemes during various speed operations. The experimental results indicated that the proposed adaptive supervisory sliding FCMAC outperformed the other two controllers under all operating conditions.

**Table 3:** The statistics of the RMSE results.

	Speed Reference	1200 rpm	2000 rpm	36 rpm	±1200 rpm
Control Scheme					
Adaptive supervisory sliding FCMAC		4.78	4.3	0.78	4.7
Adaptive sliding FCMAC		5.24	4.56	1.04	5.99
Adaptive sliding CMAC		10.9	7.23	3.33	10.41

## 5. Conclusions

This study developed an adaptive supervisory sliding FCMAC and successfully implemented it in a practical sensorless vector-controlled IM drive system. The stability of the proposed adaptive supervisory sliding FCMAC was derived using the Lyapunov approach, which guarantees learning-error convergence. The practicability and robustness of the proposed adaptive supervisory sliding FCMAC was demonstrated through simulation and experimentation. The simulation results indicated that the proposed adaptive supervisory sliding FCMAC was affected only slightly when the parameters *J_m_* and/or *B_m_* were varied. The experimental results indicated that the proposed adaptive supervisory sliding FCMAC exhibited excellent approximation and learning capabilities that were superior to the other controllers. In addition, the RMSE results further indicated that the proposed adaptive supervisory sliding FCMAC outperformed the other two controllers, confirming the effectiveness and robustness of this scheme for real IM drives.
